# Anticoagulant rodenticide poisoning in farmed Patagonian mara (*Dolichotis patagonum*)

**DOI:** 10.1186/s12917-024-03943-x

**Published:** 2024-03-07

**Authors:** Piyarat Chansiripornchai, Sawang Kesdangsakonwut, Somporn Techangamsuwan

**Affiliations:** 1 Department of Veterinary Pharmacology, Faculty of Veterinary Science, Chulalongkorn University, Henri - Dunant Road, Pathumwan, Bangkok, 10330 Thailand; 2 Department of Pathology, Faculty of Veterinary Science, Chulalongkorn University, Henri - Dunant Road, Pathumwan, Bangkok, 10330 Thailand; 3 Animal Virome and Diagnostic Development Research Unit, Faculty of Veterinary Science, Chulalongkorn University, Henri - Dunant Road, Pathumwan, Bangkok, 10330 Thailand; 4 Wildlife, Exotic and Aquatic Animal Pathology Center of Excellence, Faculty of Veterinary Science, Chulalongkorn University, Bangkok, 10330 Thailand

**Keywords:** Animal food, Contamination, Patagonian mara, Poisoning, Rodenticide

## Abstract

**Background:**

Anticoagulant rodenticide (AR) poisoning was diagnosed in 3 Patagonian maras (*Dolichotis patagonum*) raised in the mara farm in Thailand. To date, there have been no reports of maras with diagnosed AR poisoning.

**Case presentation:**

The first clinical sign of the sickening maras was anorexia. Fifteen from 50 maras were dead over a 3–5 day period after the clinical signs had occurred. Positive results to AR were detected in all of the maras’ liver specimens by screening test using thin layer chromatography and spectrophotometry methods. Supportive therapy was selected for the treatment of the 35 surviving maras. During the follow – up observation period of 12 months, all of the surviving maras were healthy and no reproductive loss.

**Conclusions:**

This is the first report on suspected AR poisoning in maras in Thailand based on history taking, clinical signs, gross pathology lesions and chemical analysis. AR poisoning in the present report is possibly from contaminated animal food. Therefore, quality control of food should be fastidious when feeding maras.

## Background

**Fig. 1:**
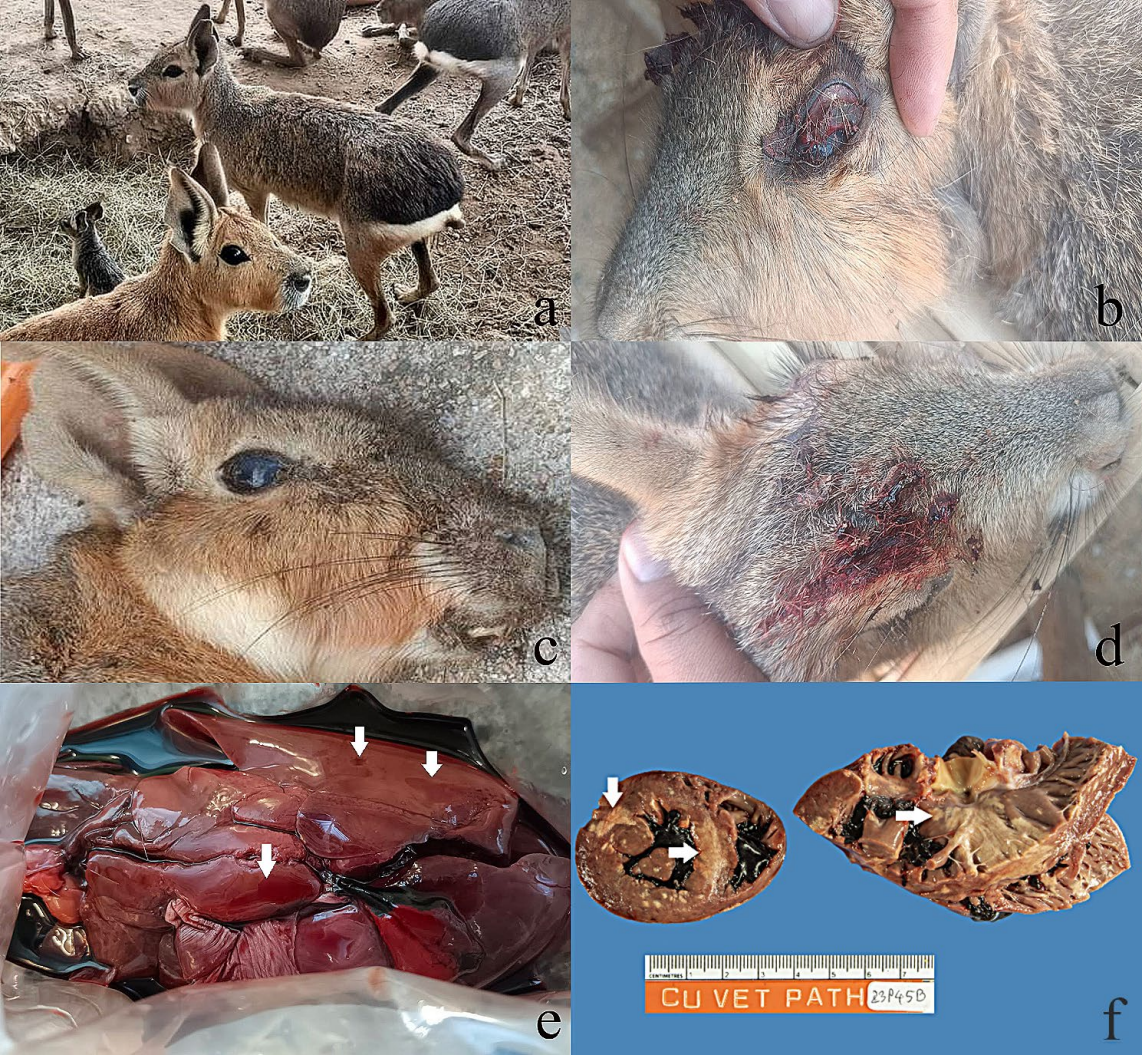
(a) Patagonian maras raised in the farm. (b) Ocular hemorrhage in the sick mara. (c) Corneal opacity in the sick mara. (d) Subcutaneous bleeding in the sick mara. (e) Macroscopic lesion of hemorrhage (arrows) in the mara’s liver specimen. (f) Multiple myocardial necrosis and calcification (arrows)

Rodents are the most numerous order of mammals. The Patagonian mara (syn. Patagonian cavy; *Dolichotis patagonum*), is a mammal that belongs to the order *Rodentia* and family *Caviidae* [1]. It is a large hystricomorph rodent closely related to the guinea pig (*Cavia porcellus*) and capybaras (*Hydrochoerus hydrochoeris*). However, in contrast to these species it is highly cursorial, occupying a niche in the Patagonian steppe resembling small antelopes or gazelles in the African savanna and Argentina. Maras weigh between 7 and 9 kg and have an external length of approximately 610–810 mm [2]. They are distinguished mainly by their long ears and longer limbs compared to other cavies. In general, Patagonian maras are characterised by an irregularly barred grey pattern of the dorsal fur, with a characteristic white patch on the rump separated from the dorsal fur by a thin black line (Fig. 1a) [3]. Maras are diurnal herbivorous hindgut fermenters who consume a variety of plants species, with a particular focus on grasses [4]. Due to their hardiness in temperate climates, maras are often displayed as semi – free - ranging animals in zoological gardens, where they can roam pastures freely or be kept in large pasture enclosures [2]. Recently, maras have become one of the popular pets for exotic animal lovers, while in the past they were not recommended to keep as pets because of their gazelle - like, nervous character [2].

In general, maras are healthy animals and easy to raise. However, maras may have health problems from infectious or non – infectious diseases. For infectious diseases, toxoplasmosis, bacterial and fungal infections have been reported as the cause of death in the maras [1]. For non – infectious diseases, maras may suffer from different types of bone fractures because they have the instincts of wildlife as part of their skills for predation by running and jumping in the air [2, 3]. Moreover, toxicosis from toxicants such as rodenticides, insecticides and herbicides are another non – infectious cause of death in exotic animals in the family *Caviidae* such as guinea pigs and maras [5].

In Thailand, there are some maras farms for pet animals in many parts of the country, i.e. Chachoengsao and Ratchaburi provinces. Normally, maras are fed with rabbit food, guinea pig food, grasses, hay, fruit and vegetables. Although maras can eat many types of food, the sources of this food should be selected carefully. Especially, contamination by toxicants such as anticoagulant rodenticides (ARs) in animal food during keeping in food storages can be harmful to maras. ARs are pesticides widely used to protect buildings, installations, crops, stored human and animal feed as well as to prevent the spread of rodent vector disease agents [6]. ARs are usually commercially available as colored soft bait or paraffin blocks containing sucrose, meat, vegetables or fruit, designed to attract rodents but may also attract non - target animals, such as pets, livestock and wild animals, causing accidental or intentional animal poisoning [7].

ARs can be divided into the first generation, such as warfarin, coumatetralyl and diphacinone and the second generation, such as bromadiolone, brodifacoum and difenacoum; the second generation of ARs has a higher binding affinity to hepatic tissues, therefore expresses higher accumulation, persistence and toxicity [8]. Both generations of ARs have a similar mechanism of action by inhibiting the activity of vitamin K epoxide reductase resulting in a progressive reduction in the pool of vitamin K required for the activation of coagulation factors II, VII, IX, and X and leading to vitamin K depletion and risk of bleeding [9]. The animals suffering from ARs intoxication always show signs of generalised bleeding. Hemorrhage secondary to ARs may occur at any site, such as the body cavity, stomach, uterus, upper airway, pericardium, joints and eyes and is potentially fatal in the absence of appropriate therapy [10]. Although black rats (*Rattus rattus*) and brown rats (*Rattus norvegicus*) are the major target species for ARs, poisoning in non – target species by accidental or intentional contact with ARs is a major concern worldwide [11]. Therefore, it is important to study and collect data warning of ARs intoxication that can be harmful to any species of animals.

The purpose of this report is to describe 3 cases of fatally intoxicated Patagonian maras with diagnosed ARs toxicity which is the first report in Thailand.

## Case description

Liver specimens from three 1 – year – old, female Patagonian maras raised in the mara farm in Chachoengsao province were submitted to the Department of Veterinary Pharmacology, Faculty of Veterinary Science, Chulalongkorn University (DVPCU) for analysis of ARs exposure. DVPCU has been one of the toxicological centers that provides determination of toxicants such as rodenticides, insecticides, herbicides and mycotoxins for animal hospitals, veterinarians, general practitioners and the public in Thailand since 1990.

From history taking, there were 50 maras raised on the farm and 15 maras died within 3–5 days after clinical signs had occurred in January, 2023. All of the 50 maras were housed both indoors and outdoors in the farm field. The owner declared that the maras had previously been well, eating, drinking and behaving normally. They were normally fed with a commercial rabbit or guinea pig food, grass, hay, as well as fresh fruit and vegetables. However, a week before the first clinical sign onset, they were fed with the rabbit food bought from a new shop that usually uses rodenticide in food storage. The initial symptoms of the sickening maras observed by the owner was anorexia, followed by ataxia, hematemesis, hematochezia, lethargy, tremors, seizures, subcutaneous bleeding, ocular hemorrhage, corneal opacity and death over a 3–5 day period (Fig. 1b–d).

The clinical signs in some of the surviving maras were anorexia and ataxia. Gross corneal opacity was observed in all maras. The livers of 3 mara specimens showed hemorrhagic lesions on gross findings (Fig. 1e). Multiple myocardial necrosis and calcification was noticed in one mara (Fig. 1f).

For chemical investigation of ARs, the maras’ liver specimens were analysed using the thin layer chromatography (TLC) technique and spectral analysis by derivative spectrophotometry [12]. Briefly, the liver specimens were ground and extracted with chloroform in a vaporous condition. The extracts were filtered and the residual material was re - extracted and re - filtered. The residue was reconstituted in 1 ml chloroform. TLC separation was performed using silica gel G plates as the stationary phase and methyl ethyl ketone:benzene (6:120, v/v) in the mobile phase. Standard solutions and control extracts of the animal samples were also prepared. Quantification was performed by spiking extracts of the animal samples with rodenticide standards, running them under the standard TLC protocol. For spot detection of ARs, a hydrogen peroxide (H_2_O_2_) solution was oversprayed on plates and then an iron (III) chloride (FeCl_3_) solution was sprayed. AR positivity was recorded when both TLC and spectrophotometry methods showed positive result.

## Discussion and conclusions

It is important to understand the health and diseases of maras, a large rodent closely related to guinea pigs, for their captive management as well as the conservation of the wild population [1]. Until now, current literature and data on the maras has been very limited and it remains unknown how sensitive maras are to ARs poisoning [3]. ARs are known for the most common poisons used to control rodents in domestic, municipal, agricultural and conservation settings but they can be occasionally harmful to non - target species, such as birds, reptiles, carnivores, lagomorpha and guinea pigs [5, 13].

**Fig. 2:**
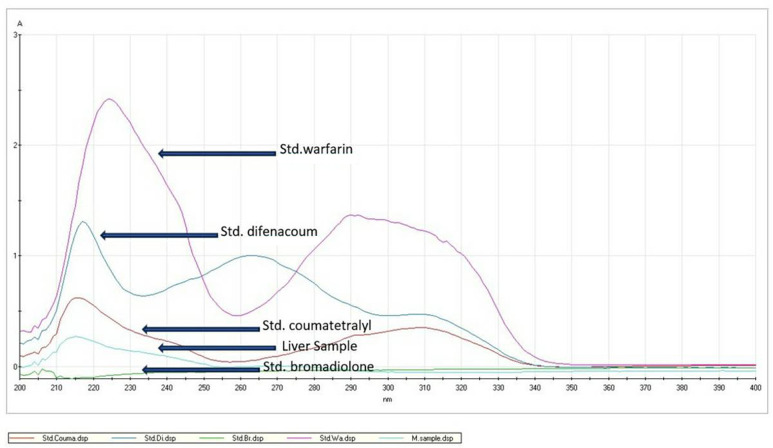
Spectrophotometry data of standard ARs and the liver specimen of a mara. X - axis: optical density (OD). Y - axis: wavelength (nanometers). Arrows show the peak points of ARs standards and liver specimens

In our present case report, ARs intoxication was detected in the liver specimens of 3 maras. By chemical determination with TLC and spectrophotometry methods, we found positive results to ARs poisoning in the liver specimens of all the 3 maras (Fig. 2). An ARs screening method such as TLC is a valuable tool for confirming the diagnosis of ARs intoxication in cases where ingestion was not witnessed. ARs screening does not allow for misreading since there are no acceptable concentrations of ARs in animals, even if the owner declares the exposure to ARs impossible [14]. Therefore, detecting positive results means that the animal has had exposure to some quantity of ARs. However, the limitations of the TLC technique include its inability to identify the specific type and measure the amount of ARs in each affected specimen, thus higher specific and more sensitive methods such as high performance liquid chromatography (HPLC) and gas chromatography mass spectroscopy (GCMS) are more suitable methods for identifying the specific type and quantity of ARs intake. Anyway, TLC and spectrophotometry methods are a reliable screening test for ARs [12].

The source of ARs detected in our cases not certainly known but may be contamination from animal food. From history taking, the maras’ owners reported that they recently raised the maras for a week with the rabbit food bought from a new shop that usually uses rodenticide in food storage. Probably, this was the route of ARs intoxication in the maras. In addition, all of the maras lived in a restricted area; therefore, they were probably exposed to ARs by simple ingestion of contaminated food in the farm. Our results indicate that use of ARs may lead to unintentional poisoning of non - target animals. Recent reports have also shown that use of ARs has resulted in environmental contamination of ARs residues and consequent mortality or morbidity in non - target species [13].

Furthermore, the clinical signs of the sick maras in our report correlated to the common clinical signs of ARs intoxication including anorexia, lethargy, lameness and ataxia. Especially, the first visible sign of ARs poisoning in rodents is significant anorexia [6]. Moreover, other less common clinical symptoms due to ARs accidental animal exposure are weakness, lethargy, coughing, vomiting, melena, paleness, dyspnea, bleeding from the gums, seizures, bruising, shaking, abdominal distention and pain [9]. In addition, liver hemorrhages and bleeding disorders (i.e. orbital hemorrhage, hematemesis and hematochezia) were detected in all 3 of the maras. Myocardial necrosis contributes as a cause of death as seen in humans that are affected with ARs induced myocardial infarction [15]. This finding correlates to the gross pathology of ARs intoxication [9, 16]. Recent reports on ARs poisoning in guinea pigs have revealed ocular lesions, i.e. exophthalmos, while ocular hemorrhage and corneal opacity has been found in the maras in our report. Therefore, ocular abnormality may a sign of ARs poisoning in family *Caviidae* as well as in dogs [5, 16, 17]. For the treatment of 35 surviving maras, the owners gave multivitamins in drinking water and observed the clinical signs closely during waiting for ARs investigation under the consult of a veterinarian. Anorexia is the unique clinical sign in the surviving maras and there were no further deaths after the death of 15 maras. After several days, the surviving maras were slightly recovered. The owners raised them with fresh and clean vegetables and fruits. During a follow – up period of 12 months, all of the surviving maras were healthy with no loss of productivity, although some still exhibited anorexia.

In conclusion, this case report described 3 Patagonian mara specimens that were diagnosed with ARs poisoning based on history taking, clinical signs, gross pathology lesions and chemical analysis using TLC and spectrophotometry methods. The results indicate that ARs contamination in animal feed is a possible cause of mortality in maras. Therefore, quality control of food should be meticulous when feeding maras.

## Data Availability

All data generated or analysed during this study are included in this published article.

## References

[1] Ostevik L, Tysnes KR, Klevar S, Debenham JJ. *Toxoplasma Gondii* infection in two captive patagonian maras. J Vet Diag Invest. 2019;31:875–8. 10.1177/1040638719883191 PMC6900725 31646951

[2] Kessler DS, Hope K, Maslanka M (2009). Behavior, nutrition, and veterinary care of Patagonian cavies (*Dolichotis patagonum*). Vet Clin North Am Exot Anim Pract.

[3] Rojas V, Fitzmaurice M, Po E, Freeman P (2023). Surgical stabilization of a traumatic comminuted vertebral fracture / luxation in a Patagonian mara (*Dolichotis patagonum*). Vet Rec Case Rep.

[4] Puig S, Cona MI, Videla F, Mendez E (2010). Diet of the mara (*Dolichotis patagonum*), food availability and effects of an extended drought in northern Patagonia (Mendoza, Argentina). Mamm Biol.

[5] Colon V, Bates N (2019). Suspected rat bait poisoning in a group of pet guinea pigs. J Exot Pet Med.

[6] Fisher P, Campbell KJ, Howald GR, Warburton B (2019). Anticoagulant rodenticides, islands and animal welfare accountancy. Animals.

[7] Gallocchio F, Basilicata L, Benetti C, Angeletti R, Binato G (2014). Multi - residue determination of eleven anticoagulant rodenticides by high - performance liquid chromatography with diode array / fluorimetric detection: investigation of suspected animal poisoning in the period 2012–2013 in north - eastern Italy. Forensic Sci Inter.

[8] Watt BE, Proudfoot AT, Bradberry SM, Vale JA (2005). Anticoagulant rodenticides. Toxicol Rev.

[9] Hovda L, Brutlag A, Poppenga R, Peterson K (2016). Rodenticides. Small animal toxicology.

[10] Stroope S, Walton R, Mochel JP, Yuan L, Enders B (2022). Retrospective evaluation of clinical bleeding in dogs with anticoagulant rodenticide toxicity - A multi – center evaluation of 62 cases (2010–2020). Front Vet Sci.

[11] Caloni F, Cortinovis C, Rivolta M, Davanzo F (2016). Suspected poisoning of domestic animals by pesticides. Sci Total Environ.

[12] Kaewamatawong T, Lohavanijaya A, Charoenlertkul P, Srichairat S (2011). Retrospective histopathological study of hemorrhagic lesion of coumarin intoxication in dogs. Thai J Vet Med.

[13] Nakayama SM, Morita A, Ikenaka Y, Mizukawa H, Ishizuka M (2018). A review: poisoning by anticoagulant rodenticides in non - target animals globally. J Vet Med Sci.

[14] Waddle LS, Poppenga RH, Drobatz KJ (2013). Anticoagulant rodenticide screening in dogs: 123 cases (1996–2003). J Amer Vet Med Assoc.

[15] Tam KW, Chan CK, Liu S (2021). Anticoagulant rodenticide ingestion: who will develop coagulopathy ?. Hong Kong J Emerg Med.

[16] Griggs AN, Allbaugh RA, Tofflemire KL, Ben-Shlomo G, Whitley D, Paulsent ME (2016). Anticoagulant rodenticide toxicity in six dogs presenting for ocular disease. Vet Ophthalmol.

[17] Brei C, Stern L, Racette M. An atypical presentation of anticoagulant rodenticide toxicosis in a dog. Can Vet J. 2023;64:1015–20. PMC10581352 37915786

